# Insights into the Impact of Low-Dose Ionizing Radiation on Neurodegenerative Disease Progression in In Vivo Models

**DOI:** 10.3390/ijms27083368

**Published:** 2026-04-09

**Authors:** Valeria V. Goloborshcheva, Yana S. Kostikova, Valerian G. Kucheryanu, Sergei G. Morozov, Viktor S. Kokhan

**Affiliations:** Institute of General Pathology and Pathophysiology, 125315 Moscow, Russiaviktor_kohan@hotmail.com (V.S.K.)

**Keywords:** neurodegeneration, neuroprotection, radiotherapy, hormesis, low-dose ionising radiation, in vivo models, dementia, Alzheimer’s disease, Parkinson’s disease

## Abstract

The effective treatment of neurodegenerative diseases (NDDs), such as Alzheimer’s disease, Parkinson’s disease, and amyotrophic lateral sclerosis, remains a critical challenge in modern medicine. Given the limitations of current therapies, alternative strategies to slow neurodegeneration are urgently needed. This study presents a critical review of the current evidence regarding low-dose ionizing radiation (IR) as a promising modality for modulating neurodegenerative processes. This study examines current experimental data on the effects of low-dose IR (LDIR) on cellular protective and compensatory mechanisms, including evidence from in vivo models of NDDs. Our analysis demonstrates that LDIR enhances antioxidant activity and DNA repair, stimulates autophagy and neuroplasticity, and modulates neuroinflammatory signaling. Collectively, these findings support the hypothesis of the neuroprotective potential of LDIR, underscoring its translational viability provided that strict dosimetric guidelines are followed and individual biological responses are rigorously monitored.

## 1. Introduction

Neurodegenerative diseases (NDDs) represent a global medical and socioeconomic challenge, driven by the worldwide aging population and a progressive burden on healthcare systems. The lack of effective disease-modifying therapies and the limitations of current protocols, which focus primarily on symptomatic relief, underscore the urgent need for fundamentally new therapeutic strategies. While conventional radiation therapy is traditionally employed for malignant neoplasms and is associated with destructive effects on tumor cells, recent experimental evidence suggests that low-dose IR (LDIR) holds significant potential to counteract neurodegenerative disorders [1,2].

Over the past few decades, a substantial body of evidence has accumulated demonstrating the non-linear nature of the biological response to radiation exposure. Unlike high-dose IR (HDIR, >10 Sv), which is typically deleterious to biological tissues, LDIR is capable of inducing proliferative activity, modulating immune responses, activating repair systems, and delaying the progression of neoplastic processes [3,4,5]. This suite of adaptive reactions is frequently characterized within the framework of the radiation hormesis phenomenon [6].

The thresholds defining LDIR remain somewhat arbitrary. Traditionally, the phenomenon of radiation hormesis is considered characteristic of low-linear energy transfer (low-LET) radiation (X-rays, γ-rays, β-particles) at absorbed doses up to 100 mGy. Numerous studies have confirmed that irradiation within this range can exert protective effects, enhancing the resistance of cells and the organism as a whole to subsequent exposure to damaging doses [7,8,9]. Furthermore, it has been shown that the upper threshold for the adaptive response can reach 0.5 Gy [8]. Exceeding this limit is thought to result in the exhaustion of compensatory reserves, primarily due to the critical accumulation of DNA double-strand breaks (DSBs) that outpaces the capacity of repair systems [8,10]. Nevertheless, recent findings are significantly reshaping these perspectives. It has been established that γ-radiation can induce an adaptive response even at relatively high doses (e.g., 3 Gy) [11]. Moreover, beneficial effects on the central nervous system (CNS) have been observed following exposure to high-LET radiation, such as heavy ions (^56^Fe, 147 keV/µm, 0.5 Gy) [11,12]. It is likely that at the systemic level, tissue-wide reparative and compensatory mechanisms play a decisive role in mediating the radiation effect, whereas DSB accumulation may not serve as a sufficiently reliable marker. The capacity of LDIR to initiate repair and compensatory processes in neural tissue is currently viewed as a promising foundation for developing neuroprotective methods and disease-modifying therapies for neurodegenerative disorders [13,14].

## 2. Molecular and Cellular Mechanisms of Radiation Hormesis in the Central Nervous System

**Figure 1:**
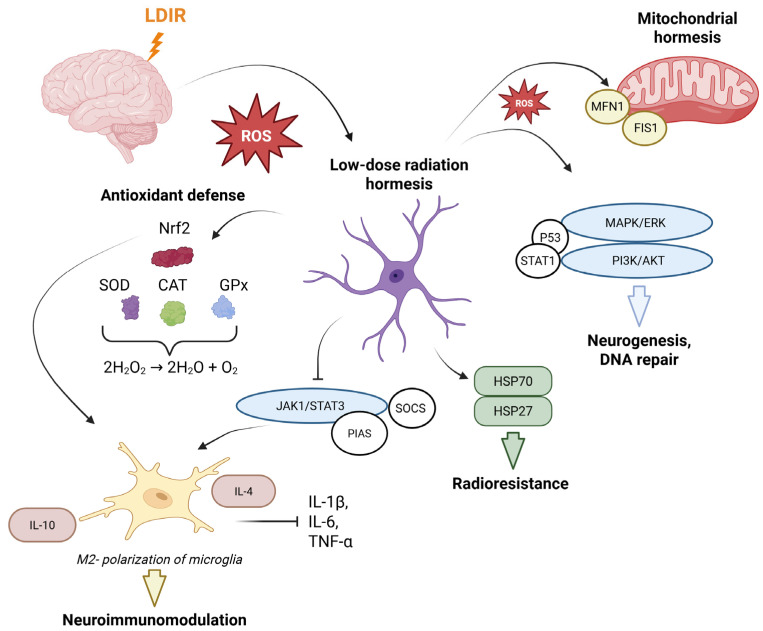
Schematic representation of the molecular and cellular mechanisms of radiation hormesis in the central nervous system. LDIR induces the generation of moderate levels of reactive oxygen species (ROS), which act as signaling molecules to initiate a cascade of adaptive responses aimed at maintaining neuronal homeostasis. Key regulatory pathways include: (1) Antioxidant response: activation of Nrf2 and subsequent induction of enzymatic defenses (SOD, CAT, GPx); (2) Mitohormesis: modulation of mitochondrial function via FIS1/MFN1 proteins; (3) Proliferation and survival: activation of the MAPK/ERK and PI3K/Akt pathways, alongside p53-mediated cell cycle regulation; (4) Neuroimmunomodulation: microglial polarization toward the anti-inflammatory M2 phenotype with a concomitant shift in the cytokine profile (increased IL-10/IL-4; decreased IL-1β, IL-6, and TNF-α); (5) Proteostasis and repair: induction of chaperones (HSP70, HSP27) and activation of the JAK1/STAT3 signaling pathway, promoting DNA repair and neurogenesis. Collectively, these processes establish a state of enhanced radioresistance and neuroprotection. Created with BioRender. Blokhin, V. (2026) https://BioRender.com/b3tdzr3 (accessed on 5 April 2026).

The impact of LDIR on the human body is multifaceted and necessitates a comprehensive analysis of its diverse effects. The biological response to LDIR is a complex, multi-level cascade governed not only by physical radiation parameters, such as the type of radiation, absorbed dose, dose rate, and exposure regimen (chronic, acute, fractionated, or combined), but also by the state of the cellular microenvironment. Together, these factors determine the integrated effect on physiological processes within target cells and tissues. In the context of neurodegeneration, a pivotal aspect of radiation hormesis is the modulation of fundamental physiological processes: i.e., a hierarchical system of interactions at the cellular, tissue, and organ levels that underpins CNS homeostasis and the organism’s adaptive capacity (Figure 1).

Evidence from in vitro and in vivo studies demonstrates that exposure to LDIR induces a complex adaptive response. This response not only mobilizes antioxidant defense systems and optimizes DNA repair processes, thereby fostering a radioresistant phenotype against subsequent high-dose irradiation [15,16,17,18], but also triggers a systemic anti-inflammatory response [5]. The latter is mediated through the repression of pro-inflammatory cytokines and the phenotypic polarization of microglia from a pro-inflammatory M1 state to a neuroprotective M2 state. Furthermore, a crucial aspect of LDIR action involves the activation of proteostasis and autophagy mechanisms, which facilitate the clearance of pathological protein aggregates, alongside the epigenetic modulation of gene expression responsible for synaptic plasticity and neuronal survival [15,16]. These mechanisms (Table 1) provide a theoretical foundation for developing therapeutic protocols aimed at slowing neuronal degradation.

### 2.1. Modulation of Redox Homeostasis and Antioxidant Defense

**Figure 2:**
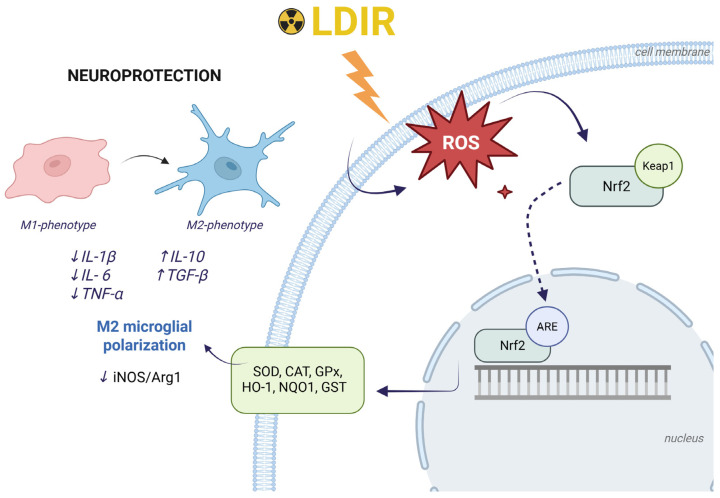
Molecular mechanisms coupling antioxidant and immunomodulatory responses under LDIR exposure. Low-dose irradiation induces the generation of moderate levels of ROS, which act as signaling molecules to trigger the dissociation of the Nrf2/Keap1 complex. Free Nrf2 then translocates to the nucleus, where it binds to antioxidant response elements (AREs), initiating the expression of detoxification and antioxidant defense genes (SOD, CAT, GPx, HO-1, NQO1, GST). Concurrently, LDIR modulates the CNS microenvironment, promoting the functional polarization of microglia from a pro-inflammatory M1 phenotype to a neuroprotective M2 phenotype (↓ iNOS/Arg1). This process is accompanied by a reorganization of the cytokine profile, characterized by decreased levels of pro-inflammatory factors (IL-1β, IL-6, TNF-α) and increased synthesis of anti-inflammatory mediators (IL-10, TGF-β). The integration of these cascades provides a comprehensive neuroprotective effect. Created with BioRender. Blokhin, V. (2026) https://BioRender.com/jl1ls8n (accessed on 5 April 2026).

Reactive oxygen species (ROS), such as the superoxide anion (O_2_^−^) and hydrogen peroxide (H_2_O_2_), are generated during redox reactions in living organisms and play a pivotal role in the development of oxidative stress associated with carcinogenesis, atherosclerosis, neurodegenerative diseases, and aging [19]. Under physiological conditions, protective cascades ensure the synthesis of key antioxidant enzymes: superoxide dismutase (SOD) catalyzes the conversion of O_2_^−^ to H_2_O_2_, which is subsequently decomposed into water and oxygen by catalase (CAT) and glutathione peroxidase (GPx). The progression of NDDs, such as Alzheimer’s and Parkinson’s diseases, is fundamentally linked to the functional degradation of the Nrf2 signaling axis, which triggers a systemic failure of metabolic homeostasis [20]. In this context, the neuroprotective potential of low-dose radiation stems from LDIR’s ability to act as a mild pro-oxidant stimulus that activates nuclear erythroid 2-related factor 2 (Nrf2)—the master regulator of the cellular antioxidant response, coordinating the expression of enzymes involved in antioxidant defense, detoxification, and repair [21] (Figure 2).

Under conditions of oxidative stress, Nrf2 is released from its cytoplasmic complex with the inhibitory Kelch-like ECH-associated protein 1 (KEAP1) [22] and translocates to the nucleus. There, it binds to antioxidant response elements (AREs) within the promoter regions of target genes, triggering the transcription of a broad spectrum of cytoprotective enzymes: heme oxygenase-1 (HO-1), NAD(P)H:quinone oxidoreductase 1 (NQO1), glutathione S-transferase (GST), as well as GPx, SOD, and CAT [23,24,25,26]. In the context of LDIR-induced radioresistance, particular importance is attributed to manganese superoxide dismutase (MnSOD)—the primary mitochondrial antioxidant that maintains bioenergetic homeostasis and establishes the basis for mitohormetic effects [27,28].

The crosstalk between the Nrf2 pathway and DNA repair mechanisms is further evidenced by findings that its activation (whether radiation-induced or chemically triggered by sulforaphane [29] or naphthoquinone [30]) promotes the attenuation of induced DSBs of DNA [30,31] and enhances radioresistance [32]. Within the CNS, the neuroprotective effect of Nrf2 is multifaceted, operating both at the neuronal level and through the modulation of the microenvironment. On one hand, Nrf2 provides direct neuronal protection by reducing radiation-induced apoptosis through the modulation of signaling pathways (specifically the Nrf2–Notch1 axis) and the mitigation of ROS levels [33,34]. On the other hand, Nrf2 acts as a systemic regulator of the neuroimmune response by inducing microglial polarization toward the anti-inflammatory M2 phenotype. This process is accompanied by the repression of pro-inflammatory cytokine production and the up-regulation of regenerative factors, such as arginase-1 (Arg-1) and CD206, which are critical for restoring CNS structural integrity in neurodegenerative conditions [35,36].

### 2.2. Neuroimmunomodulation and Systemic Anti-Inflammatory Response

The biological effects of LDIR are orchestrated through the complex modulation of immune functions, evidenced by the ability to attenuate chronic inflammation and activate cellular and humoral defense mechanisms [37]. In contrast to the deleterious effects of high doses, LDIR initiates signaling cascades aimed at maintaining cellular homeostasis [38]. The acceleration of DNA repair following irradiation is supported by a specific reorganization of the cytokine profile: the activation of IL-1, IL-3, GM-CSF, and G-CSF promotes cell viability and optimizes hematopoiesis, while low concentrations of IL-6 may serve as a survival regulator, preventing the initiation of apoptotic programs [39,40,41].

A critical aspect of LDIR action in the pathogenesis of neurodegenerative diseases is its ability to significantly downregulate the production of key pro-inflammatory mediators, including IL-1β, IL-6, and TNF-α [42]. The reduction in these factors contributes to the dampening of the systemic inflammatory response and the minimization of CNS tissue damage. This effect is closely coupled with the aforementioned functional polarization of microglia, the transition from an aggressive pro-inflammatory state (M1 phenotype) to a neuroprotective and regenerative state (M2 phenotype) [35].

Recent studies in breast cancer models, where metastasis is associated with the epithelial–mesenchymal transition (EMT) and the acquisition of a cancer stem cell (CSC) phenotype, demonstrate that the M1-to-M2 transition is largely mediated by the inhibition of the JAK1/STAT3 signaling pathway. The activation of the JAK1/STAT3 pathway serves as a key trigger for both neuroinflammation and carcinogenesis; its suppression by LDIR (regulated by SOCS and PIAS proteins) leads to a downregulation of damage markers, including a reduction in the CD44+/CD24− cell population, while simultaneously enhancing the synthesis of regenerative factors that facilitate structural tissue repair and the clearance of metabolic waste products [43]. Furthermore, additional modulation of the innate immune system under LDIR is achieved through the activation of the cGAS-STING cascade, initiated by the release of mitochondrial DNA (mtDNA) fragments into the cytosol. The mtDNA-mediated activation of the cGAS-STING pathway under LDIR conditions represents a pivotal link in non-targeted effects (NTE), ensuring ‘controlled’ immunomodulation and the induction of an adaptive response [44]. Thus, LDIR-mediated immunomodulation is a coordinated process that shifts the biological system from destructive inflammation toward a pathway of effective neuroprotection.

### 2.3. Regulation of Cellular Signaling, Proliferation, and Synaptic Plasticity

Experimental evidence indicates that LDIR acts as a precision modulator of intracellular signaling, activating cascades associated with ataxia-telangiectasia mutated (ATM) serine/threonine kinase, ERK, MAPK, c-Jun N-terminal kinase (JNK), and p53 [45,46,47]. Within the framework of radiation hormesis, these pathways mediate an adaptive response aimed at maintaining genomic stability and establishing a radioresistant phenotype. A fundamental distinction in the biological action of LDIR lies in its selectivity: low doses stimulate the proliferation of normal cells (e.g., the 2BS cell line) via the activation of the MAPK/ERK and PI3K/AKT axes, whereas such effects are not observed in tumor cells (e.g., the NCI-H446 cell line) [46]. The capacity of LDIR to optimize specific cellular functions without inducing genotoxic stress is further supported by other models; for instance, a dose of 25 mGy transiently enhances the secretory activity of pancreatic β-cells through the p38 MAPK pathway and the PDX-1 transcription factor without inducing DSB of DNA, in stark contrast to the effects of HDIR at 2.5 Gy [45,48].

In the context of neuroprotection, the most significant effect of modulating these signaling cascades is the stimulation of neural stem cell proliferation. LDIR has been shown to enhance hippocampal neurogenesis, which correlates with improved cognitive function and learning abilities in experimental models [49,50]. Transcriptomic analysis has revealed fundamental differences in cellular responses to low versus high radiation doses. While HDIR induces DNA damage response programs, cell cycle arrest, and apoptosis, LDIR predominantly activates genes responsible for antioxidant defense, MAPK/ERK signaling, glycolysis, and mitochondrial function [51]. This further confirms the role of ROS as signaling molecules that initiate metabolic adaptation.

Nevertheless, despite this pronounced neuroprotective potential, the impact of LDIR on cell cycle regulation remains an open question. In particular, the dual role of p21, a key cell cycle regulator that may determine the balance between neuronal survival and the activation of apoptotic programs, requires further investigation [47]. Given the dose-dependent nature of chromosomal damage within the 10–200 mGy range, precise control of dosimetric parameters remains a critical prerequisite for the safe translation of these effects into the therapy of NDDs.

### 2.4. Induction of Proteostasis and DNA Repair Mechanisms

A critical component of the adaptive response to LDIR is the activation of systems dedicated to maintaining proteostasis. A central role in this process is played by heat shock proteins (HSPs), a family of molecular chaperones whose expression is induced in response to thermal, oxidative, and radiation stress. The primary function of HSPs is to ensure correct protein folding, prevent aggregation, and protect cells from apoptosis, which is of paramount importance for neuronal survival [52].

Research confirms that high basal expression of HSP70 and HSP27 directly correlates with the development of a stable radioresistant phenotype. Specifically, in head and neck squamous cell carcinoma (HNSCC) lines and breast cancer side population (SP) cells (MCF-7), the levels of these chaperones increase significantly following irradiation, ensuring cell survival under radiation exposure [53,54]. For instance, it has been demonstrated in HNSCC lines that cells with elevated HSP70 content maintain higher proliferative potential when exposed to doses ranging from 2 to 12 Gy compared to normal human dermal fibroblasts (NHFs) and human dermal microvascular endothelial cells (HDMECs) [53]. A similar pattern is observed in the MCF-7 line, where the increased radioresistance of the SP subpopulation is attributed to the marked overexpression of HSP27 and HSP70 in response to a 5 Gy radiation exposure [54]. Notably, the realization of protective effects via the HSP system follows the general laws of hormesis and is characterized by a narrow ‘therapeutic window’. Analogous to thermal stress, where beneficial effects on viability are observed only within a strictly limited range of conditions [55], the efficacy of radiation-induced proteostasis in the CNS is critically dependent on the dose.

In parallel with chaperone systems, LDIR activates high-precision DNA repair mechanisms, specifically the DNA double-strand break repair (DSBR) system [56]. The central regulator of this process is the ATM kinase, which recognizes breaks and initiates a phosphorylation cascade of effector proteins. This enzyme regulates mitochondrial function, induces cell cycle arrest via p53 activation, and coordinates the choice between DNA repair and the induction of apoptosis [57]. Another critical element is poly(ADP-ribose) polymerase 1 (PARP1), which detects DNA breaks, stabilizes damaged sites, and recruits additional repair factors [58]. According to current understanding, LDIR-induced radioresistance is achieved through the synergistic activation of the aforementioned ATM kinase, PARP1, the transcription factor STAT1 [59], and the ATM/ERK/NF-κB signaling pathway [48]. Together, these components form the ‘first line of defense’ for DNA, facilitating the rapid initiation of signaling cascades that prevent lesions from progressing into lethal chromosomal aberrations. Notably, LDIR activates not only DSBR but also a broad spectrum of genome surveillance systems, including mismatch repair (MMR). For instance, a significant increase (up to five-fold) in the expression of DNA repair genes (*hMSH2* and *hMSH6*) has been observed in medical personnel with prolonged occupational exposure to LDIR [60]. Thus, adaptation to radiation exposure is characterized by a dose-dependent and cumulative nature, enhancing the efficiency of quality control systems for both protein integrity and genetic stability.

### 2.5. Mitohormesis and Metabolic Remodeling

The phenomenon of mitohormesis, triggered by exposure to LDIR, represents an adaptive restructuring of the mitochondrial network aimed at restoring the bioenergetic capacity of neurons and maintaining a dynamic equilibrium between biogenesis and mitophagy. Mitochondrial dysfunction, manifested by impaired energy metabolism and exacerbated oxidative stress, is a cornerstone of the pathogenesis of major neurodegenerative diseases [61]. Paradoxically, the moderate oxidative stress induced by LDIR acts as a signaling stimulus that activates compensatory systems through mitochondrial adaptation mechanisms, whereas HDIR causes direct cellular damage [62,63,64,65].

A central role in mediating this response is played by the activation of the SIRT1/PGC-1α axis, which coordinates the expression of nuclear and mitochondrial genes responsible for organelle biogenesis and the efficiency of oxidative phosphorylation [66,67]. LDIR stimulates the transcription of respiratory chain components and modulates the activity of the integral outer mitochondrial membrane mitochondrial fission protein 1 (FIS1) and Mitofusin-1 (MFN1), which are essential for maintaining organelle integrity [62].

A crucial aspect of metabolic remodeling is the enhancement of the protein and organelle quality control systems. Hormetic radiation doses likely facilitate the timely clearance of damaged mitochondria via Pink1/PARK2/mROS-dependent mitophagy, thereby preventing excessive ROS generation and the initiation of apoptotic cascades [68,69]. Thus, the dual role of ROS under LDIR exposure lies, on one hand, in triggering cytoprotective pathways (such as the aforementioned Nrf2/ARE) that elevate cellular antioxidant status, and on the other, in maintaining signaling oxidation levels required for the selective removal of irreversibly damaged organelles, thereby ensuring neuronal metabolic plasticity. The stimulation of mitohormesis represents a promising neuroprotective strategy that promotes the long-term reinforcement of bioenergetic homeostasis in the CNS and delays neurodegeneration [70].

**Table 1:** Interplay between intracellular adaptive mechanisms and systemic biological effects induced by low-dose ionizing radiation.

Protective and Adaptive Mechanisms	Key Markers and Signaling Pathways	Biological Effects	References
Antioxidant Defense	Nrf2/ARE pathway, SOD, MnSOD, CAT, GPx, HO-1, NQO1, GST	ROS neutralization, reduction in oxidative stress, neuronal protection against apoptosis	[27,28,30,32,33]
Neuroimmunomodulation	↓iNOS/Arg1, ↓IL-1β, ↓IL-6, ↓TNF-α, ↑IL-10, ↑TGF-β, ↑T-cell response	Mitigation of chronic neuroinflammation, establishment of a regenerative CNS microenvironment	[35,36,39,40,41,42,71]
Proliferation and Neuroplasticity	MAPK/ERK pathway, PI3K/Akt, p53, p38/MAPK/PDX-1 pathway	Stimulation of hippocampal neurogenesis, improvement of cognitive functions and synaptic transmission	[45,46,47,48,49,50,51]
Proteostasis and DNA Repair	HSP70, HSP27, ATM kinase, PARP1, STAT1, ATM/ERK/NF-κB, *↑hMSH2* and *↑hMSH6*, NPY, PCP4	Correct protein folding, prevention of protein aggregation and proteopathy, restoration of genomic integrity	[53,54,56,59,60,72]
Mitohormesis	SIRT1/PGC-1α pathway, FIS1/MFN1, Pink1/PARK2 pathway *	Restoration of neuronal bioenergetics, clearance of damaged mitochondria (mitophagy)	[62,66,67,68,69]

* Hypothetically; ↓ decrease; ↑ increase.

## 3. Neurobiological Effects of Low-Dose Ionizing Radiation in Experimental In Vivo Models of Neurodegenerative Diseases

The application of the radiation hormesis concept within the context of neurodegenerative diseases offers unique opportunities to modulate pathological processes by stimulating endogenous defense systems. Unlike high doses that induce overt tissue damage, LDIR acts as a moderate stressor, triggering a cascade of adaptive neurobiological reactions, as evidenced by numerous experimental studies [73,74]. A pivotal mechanism in this process is the activation of programs responsible for maintaining cellular homeostasis [65]. Particularly within the doses around 100 mGy, experimental data have further confirmed that LDIR can induce epigenetic modifications that regulate the expression of antioxidant defense and repair genes [75], as well as influence neuronal differentiation [70], thereby contributing to an increase in overall lifespan [76].

The capacity of LDIR to modulate the structural and functional plasticity of the brain has become a focal point of in vivo research. It has been established that low radiation doses can stimulate the proliferation of neural stem cells and hippocampal neurogenesis [50], as well as normalize synaptic transmission through the modulation of neurotrophic factor levels, such as BDNF and NGF [77]. Collectively, these effects, particularly those framed within the context of radiation hormesis, establish a biological foundation for the neuroprotective potential of LDIR. This potential is further substantiated by observed improvements in cognitive function and the attenuation of neurodegeneration across various experimental models of NDDs discussed hereafter (Table 2).

### 3.1. Effects of LDIR in Experimental Models of Alzheimer’s Disease

The application of the radiation hormesis concept in the therapy of Alzheimer’s disease (AD) is based on the ability of LDIR to modulate key pathogenic pathways: the accumulation of amyloid-beta (Aβ) aggregates, the formation of microtubule-associated protein tau neurofibrillary tangles, and the development of chronic neuroinflammation [78].

In vivo experimental studies demonstrate the significant therapeutic potential of LDIR in reducing amyloid burden. A seminal study by Marples et al. first demonstrated that localized cranial irradiation promotes a reduction in amyloid plaque count in transgenic *B6.Cg-Tg(APPswe,PSEN1dE9)85Dbo/J* mice [79]. Mechanistically, this effect may be attributed to both direct physicochemical impact, such as the disruption of hydrogen bonds within the β-sheet structure of amyloid fibrils and the depolymerization of glycosaminoglycans [14,80], and the activation of biological clearance systems. Specifically, in the 5xFAD mouse model, it was established that fractionated irradiation (total dose 10 Gy, 2 Gy/fraction) induces the expression of the triggering receptor expressed on myeloid cells 2 (TREM2) on microglia, facilitating a phenotypic shift from a pro-inflammatory M1-like state to a phagocytic M2-like phenotype [36]. This transition is accompanied by a decrease in pro-inflammatory cytokine levels (e.g., TNF-α) and an increase in anti-inflammatory factors (e.g., TGF-β), correlating with improved spatial memory in the subjects [36,81]. Further evidence of the multi-target nature of such exposure (within the 0.5–2 Gy dose range) is provided by data showing the concomitant reduction in both amyloid burden and phosphorylated tau levels, which collectively ensures robust neuroprotection [82].

Current evidence suggests that even complex combined irradiation (γ-rays and ^12^C carbon ions) exerts a regulatory influence on the immune system. In 5xFAD transgenic mice, such exposure led to a “rebalancing” of the cytokine profile in the prefrontal cortex and hippocampus, alongside improved odor recognition memory. Furthermore, 3.5 months post-irradiation, these mice exhibited increased levels of macrophage inflammatory protein-1α (MIP-1α) in the prefrontal cortex and a reduction in IL-2β levels in the hippocampus [81].

The neuroprotective effects of radiation also effectively attenuate tau pathology. Experimental studies using the 3xTg-AD line (*B6;129-Tg(APPSwe,tauP301L) 1LfaPsen1tm1Mpm*) demonstrated that a course of right-hemisphere brain irradiation (2 Gy for 5 days) results in a concurrent reduction in Aβ-fractions and phosphorylated tau protein levels [83]. Similarly, in the Tau P301S homozygous model, combined irradiation (0.24 Gy γ-rays and 0.18 Gy ^12^C) significantly improved locomotor function and endurance while exerting an anxiolytic effect [84]. This progression is associated with pronounced modulation of microglial activity and the upregulation of cytokines in the hippocampus and cerebellum, indicating the activation of adaptive CNS resources in response to combined radiation stress in irradiated animals [84]. Additionally, LDIR stimulates regeneration via the induction of vascular endothelial growth factor (VEGF) and growth-associated protein 43 (GAP-43), supporting axonal growth and myelination [85].

However, there are grounds to believe that the efficacy of LDIR may be limited by sexual dimorphism. In the TgF344-AD rat model, it was established that females are less sensitive to the anti-amyloid effects of IR (2 Gy for 5 days), despite a decrease in microglial inflammatory markers (CD68) [80]. This underscores the critical importance of personalizing radiation protocols to achieve the desired biological response.

The translational potential of these findings is supported by clinical observations. Pilot trials conducted at the University of Virginia in 2023 confirmed the safety of fractionated irradiation (2 Gy for 5 days) in patients with AD and its ability to slow cognitive decline [86]. Cases of low-dose CT (40–80 mGy) application also indicate the possibility of restoring basic functions in patients with severe dementia [86,87]. Despite these promising results, these data require verification in large-scale randomized controlled trials.

### 3.2. Impact of LDIR on Pathogenetic Targets in In Vivo Models of Parkinson’s Disease

Recent experimental studies highlight the multi-level neuroprotective effects of LDIR in Parkinson’s disease (PD) models. LDIR exposure emerges as a promising strategy for PD, aimed at activating preconditioning stimuli and enhancing the endogenous defense mechanisms of dopaminergic neurons.

A key mechanism underlying the hormetic response in PD is the effective suppression of oxidative stress and neuroinflammation. In studies utilizing classical MPTP-induced models, LDIR exposure (specifically, 0.5 Gy total-body irradiation) was found to induce a rapid surge in antioxidant enzyme activity (CAT and GSH) as early as 3 h post-exposure [74]. Furthermore, it has been demonstrated that LDIR significantly reduces the intensity of oxidative stress and the severity of apoptosis in neurons of the substantia nigra pars compacta and striatum. These findings are complemented by contemporary research confirming that low doses (approximately 0.6 Gy) exert anti-inflammatory effects by suppressing the expression of pro-inflammatory and glial activation markers in the striatum (e.g., GFAP, CD54) [88].

Of particular interest is the selective impact of LDIR on key molecular targets associated with the disease. Studies in large mammals (minipigs) revealed a specific reduction in LRRK2 protein levels in the striatum 28 days post-irradiation, a critical genetic risk factor for PD [89]. Notably, under these irradiation protocols (cumulative dose < 2 Gy), the levels of other proteins, including α-synuclein, PARK2, and tyrosine hydroxylase, remained stable, with no detectable pathological changes, vascular lesions, or reactive gliosis in the brain tissue [90]. Such selectivity suggests the potential for targeted intervention on specific molecular pathways without systemic disruption of neuronal metabolism, further confirming the existence of a safe “therapeutic window” for LDIR application.

Beyond targeted monotherapy, combination therapy represents a promising avenue for PD treatment, demonstrating pronounced synergistic effects. For instance, the combination of *Ginkgo biloba* extract (EGb761) and fractionated low-dose γ-irradiation (0.25 Gy weekly for 6 weeks; total cumulative dose of 1.5 Gy) in a reserpine-induced parkinsonism model not only restored striatal dopamine levels but also normalized antioxidant system markers, including GSH, MDA, and iron ion concentrations [91]. These integrated approaches provide neuroprotection by addressing mitochondrial dysfunction and stabilizing cellular homeostasis. Consequently, LDIR emerges as a promising tool for developing innovative PD therapeutic strategies, capable of slowing degenerative processes through the stimulation of adaptive CNS resources and the selective modulation of target proteins.

### 3.3. Experimental Evidence and Prospects for Modulating Amyotrophic Lateral Sclerosis Pathogenesis via LDIR

Amyotrophic lateral sclerosis (ALS) is a progressive NDD characterized by the selective loss of motor neurons, leading to paresis, progressive muscular atrophy, and terminal respiratory failure [92]. Unlike Alzheimer’s and Parkinson’s diseases, research into the effects of LDIR on experimental ALS models is in its infancy; however, preliminary data suggest a complex and non-linear nature of this interaction.

In a recent fundamental in vivo study using a minipig model, it was demonstrated that a single dose of total-body irradiation (1.79 Gy) can modulate the expression and intracellular distribution of key ALS-associated proteins, including FUS/TLS, C9orf72, STMN2, and pTDP-43 [93]. Notably, the observed changes were strictly tissue- and cell-specific, varying across different brain regions and cellular compartments (nucleus vs. cytoplasm). The authors emphasize that these molecular shifts cannot be unequivocally interpreted as the activation of a pathological cascade; rather, they may represent an adaptive response or a hormetic effect that could exert neuroprotective actions in certain contexts. These findings open a new avenue in ALS etiology research, suggesting that low-intensity external radiation factors may potentially influence the molecular triggers of the disease.

In contrast to fundamental research on pathogenesis, clinical application of radiotherapy in ALS is currently limited to palliative care and does not aim to modify the neurodegenerative process. Low-dose irradiation of the salivary glands is a recognized clinical practice to manage sialorrhea in ALS patients, providing a temporary improvement in quality of life [94]. This therapeutic approach remains purely symptomatic and does not target the underlying pathogenetic mechanisms. Consequently, further investigation into dose-dependent effects and radiation hormesis mechanisms in animal models of ALS represents a promising research direction. Understanding the conditions under which LDIR triggers adaptive protective responses (aimed at preserving respiratory and locomotor functions) versus those that may promote the aggregation of pathological proteins (e.g., pTDP-43, FUS/TLS) could be key to developing novel preventive or therapeutic strategies to maintain neuromuscular functionality.

### 3.4. Potential Applications of LDIR in Traumatic Injuries of the Nervous System

Experimental studies across various models demonstrate a broad spectrum of neuroprotective effects of LDIR that extend beyond classical neurodegenerative models. For instance, in a model of retinitis pigmentosa (rd10 mice; *B6.CXB1-Pde6brd10/J*), LDIR at an absorbed dose of 0.65 Gy exhibited a pronounced protective effect, significantly delaying photoreceptor cell death and restoring functional activity. The molecular mechanism underlying this protection is associated with the activation of the antioxidant enzyme gene peroxiredoxin-2 (Prdx2), with a prolonged therapeutic effect achieved through repeated irradiation protocols [95]. Of particular significance are data obtained from models of peripheral nervous system injuries. It has been established that l irradiation at a total dose of 7 Gy following sciatic nerve injury in rats stimulates regenerative processes, suppresses fibrous scar formation, and significantly improves electrophysiological parameters of conduction recovery [96].

A critical aspect of these effects is systemic immunomodulation. LDIR has been shown to optimize neural tissue repair by activating T-cell-mediated immunity and modulating associated cytokine profiles; notably, the absence of a regenerative response in immunodeficient animals confirms the systemic nature of this adaptation [71]. Furthermore, the modulation of neuroprotective factors, such as neuropeptide Y (NPY) and Purkinje cell protein 4 (PCP4), identified in intact swine, suggests that LDIR can induce a state of preconditioning, potentially increasing CNS resistance to subsequent neurodegenerative and behavioral impairments [72].

In conclusion, the gathered evidence confirms the universal nature of the radiation hormesis phenomenon within the nervous system. The ability of LDIR to activate endogenous repair mechanisms and proteostasis, among other effects, opens new perspectives for developing comprehensive rehabilitation strategies for both traumatic and degenerative conditions of the CNS and peripheral nerves.

**Table 2:** Neuroprotective effects of LDIR in experimental models of NDDs and traumatic CNS injuries in vivo.

Pathology	Subject (Model)	Irradiation Regimen	Key Biological Effects	References
Alzheimer’s disease	Mice (5XFAD, 3xTg-AD, APPswePSEN1)	γ-rays 0.5–2 Gy (fractionated); γ-rays 0.24 Gy + ^12^C nuclei 0.18 Gy (combined)	Reduction in amyloid burden (↓Aβ); M1→M2 microglia polarization (TREM2); increased neuronal viability; ↑VEGF, ↑GAP-43; improvement of cognitive functions	[81,82,84,85]
Parkinson’s disease	Mice (MPTP); rats (reserpine); minipigs	γ-rays 0.5–0.6 Gy (single dose); γ-rays 0.25 Gy × 6 weeks (cumulative dose 1.5 Gy)	Activation of CAT and GSH; ↓LRRK2 in the striatum; suppression of neuroinflammation (↓GFAP, ↓CD54); synergy with *Ginkgo biloba*—restoration of dopamine, GSH, MDA levels, and iron ions in the striatum	[74,88,89,91]
Amyotrophic lateral sclerosis	Minipigs	γ-rays 1.79 Gy (single dose)	Modulation of expression and distribution of FUS/TLS, C9orf72, STMN2, and pTDP43 proteins	[93]
Retinitis pigmentosa	rd10 mice	γ-rays 0.65 Gy (repeated)	Delayed photoreceptor cell death; activation of the antioxidant enzyme Prdx2	[95]
Sciatic nerve injury	Female Wistar rats	γ-rays 7 Gy (single dose)	Stimulation of regeneration; suppression of scarring	[96]

↓ decrease; ↑ increase; → transition.

### 3.5. Therapeutic Dose Range and Possible Side Effects of LDIR

A significant challenge in the therapeutic application of LDIR is establishing an effective dose. To our knowledge, dose-dependent effects have not been precisely studied (within a 10% step), primarily due to high costs and the bioethical constraints regarding the use of laboratory animals. Since the effects of LDIR depend on the baseline state of the CNS, results from studies on healthy brains should be interpreted with caution [11,97]. It is likely that an acute local damage, which might impair CNS functions in a physiological state, is actually necessary to trigger the neuroprotective effects observed under NDD conditions. This therapeutic effect typically manifests after a delay and can persist for up to 20 months in rodents [98,99,100,101].

Studies on γ-ray exposure demonstrate a non-linear dose-dependency. For instance, therapeutic effects were observed in a Parkinson’s disease mouse model at 2 Gy, but not at 0.5 or 3.5 Gy [102]. Other research indicates that γ-ray doses between 1 and 3 Gy suppress neurogenesis, whereas an absorbed dose of 0.3 Gy has the opposite effect [49,103,104]. Using a fractionation schedule, acute irradiation of 0.6 Gy produced a therapeutic effect with a total absorbed dose of 3 Gy; similarly, acute irradiation of 2 Gy was effective at a total dose of 10 Gy, but not 20 Gy [79,88]. While clinical case reports have shown positive outcomes at doses of 120–140 mGy, these studies did not specifically analyze dose dependence [105,106]. Consequently, while a strict therapeutic window remains undefined, we suggest the lower limit is marked by an acute dose of >0.3 Gy (the threshold for significant damage to healthy tissue), while the upper limit is guided by reports of positive NDD outcomes at ~2 Gy. Notably, the total absorbed dose under fractionation can reach 10 Gy, potentially exceeding the efficacy of acute LDIR.

Research into the therapeutic benefits of heavy charged particles remains extremely limited. Most studies have focused on the cumulative risks of particle IR and NDDs to evaluate safety for crewed space missions. The first proposal to use particle IR as a therapeutic tool for NDDs appeared in 2020 [107], by which time the non-linear effects of such irradiation were already recognized [101,108,109]. Synthesizing these data is complicated by the fact that effects depend not only on dose but also on particle mass and energy. Nevertheless, most neuroprotective effects under NDDs have been recorded at doses of 0.1–1 Gy [12,110,111].

The use of radiotherapy is associated with certain adverse effects [97,112]. For example, whole-body γ-ray exposure can reduce lifespan at 0.9 Gy, but not at 0.45 Gy [113]. Targeted head irradiation carries risks of carcinogenesis (linear increase > 0.1 Gy), cataracts (>0.5 Gy), persistent neurogenesis suppression (>5 Gy for γ-rays; 0.3–0.5 Gy for particle IR), blood–brain barrier disruption (>5 Gy), microvascular damage (>20 Gy), and retinal damage (>30 Gy) [11,114,115,116,117]. However, specialized collimators and ridge filters now enable precise targeting of specific NDD-affected brain structures [118]. This precision limits damage to healthy tissues, such as the eyes and neurogenic zones. Given that NDDs are progressive, leading to the irreversible loss of motor and cognitive functions, loss of social autonomy, and finally death, we maintain that the therapeutic benefits within these limits (2 Gy acute; 10 Gy fractionated) outweigh delayed side effects like carcinogenesis. Ultimately, LDIR therapy may represent the first-ever disease-modifying strategy for treating NDDs.

## 4. Clinical Translation Prospects of LDIR as a Systemic Modulator of Neurodegeneration

The collective evidence, including findings from in vivo models, demonstrates the complex and multifactorial neuroprotective potential of LDIR, which targets key pathogenetic nodes of NDDs. While monoclonal Aβ-antibody therapies are characterized by limited efficacy and a high risk of amyloid-related imaging abnormalities (ARIA) [119,120,121], and current treatments for PD, ALS, and other NDDs remain largely symptomatic, LDIR exposure is capable of triggering a comprehensive physiological response. These observations underscore the need to re-evaluate current therapeutic paradigms and justify the search for novel, fundamental approaches to NDD treatment.

Notably, the clinical translation of this approach has already commenced, including prospective randomized trials (e.g., *NCT05635968*). Importantly, these LDIR protocols were not associated with the serious adverse events typical of Aβ-immunotherapy and resulted in documented improvements in neurocognitive function tests [122]. Consequently, in vivo experimental data position LDIR as a foundation for a pathogenetically grounded three-stage therapeutic approach to NDDs:

*First stage:* induction of apoptosis in senescent cells and dysfunctional neurons;

*Second stage:* activation of proteome clearance systems (e.g., targeting Aβ-aggregates, tau protein, and FUS/TLS protein);

*Third stage:* stimulation of endogenous neurorepair and neurogenesis.

Naturally, the widespread clinical adoption of LDIR requires large-scale trials to confirm long-term safety and efficacy, while accounting for potential sexual dimorphism and other biological variables in treatment response [80,123,124].

## 5. Concluding Remarks and Future Directions

The synthesized in vivo data confirm that LDIR initiates a systemic adaptive response mediated through the mechanisms of radiation hormesis, including antioxidant system activation, neuroimmunomodulation, and the stimulation of neuroplasticity and proteostasis maintenance systems. The capacity of LDIR to target the fundamental pathogenetic nodes of neurodegenerative diseases positions this method not merely as a tool for symptomatic relief but as a promising instrument for the disease-modifying modulation of the neurodegenerative process.

Despite its pronounced therapeutic potential, the transition to widespread clinical practice necessitates precise standardization of dosimetric protocols, accounting for individual biological variability and sexual dimorphism. Future research should focus on elucidating the underlying nature of radiation hormesis, exploring the synergy between LDIR and pharmacological agents, and verifying neuroprotective effects through large-scale randomized controlled trials. Systematizing these approaches will establish the foundation for personalized pathogenetic strategies in nuclear medicine, aimed at the effective management of neurodegenerative disorder progression.

## Data Availability

No new data were created or analyzed in this study. Data sharing is not applicable to this article.

## Abbreviations

The following abbreviations are used in this manuscript:

2BS	Human embryonic lung diploid fibroblast cell line
A549	Adenocarcinomic human alveolar basal epithelial cell line
AD	Alzheimer’s disease
Akt (PKB)	Protein kinase B
ALS	Amyotrophic lateral sclerosis
ARE	Antioxidant response element
Arg1	Arginase-1
ATM	Ataxia-telangiectasia mutated (serine/threonine kinase)
Aβ	Amyloid-beta peptide
BDNF	Brain-derived neurotrophic factor
C9orf72	Chromosome 9 open reading frame 72 protein
CAT	Catalase
CCR2	C-C chemokine receptor type 2
CCS	Copper chaperone for superoxide dismutase
cGAS	Cyclic GMP-AMP synthase
CNS	Central nervous system
CSCs	Cancer stem cells
CT	Computed tomography
DNA	Deoxyribonucleic acid
DSB	DNA double-strand break
DSBR	Double-strand break repair
EGb761	Standardized extract of Ginkgo biloba
EMT	Epithelial–mesenchymal transition
ERK	Extracellular signal-regulated kinases
FIS1	Mitochondrial fission protein 1
FUS/TLS	Fused in Sarcoma/Translocated in Liposarcoma protein
GAP-43	Growth-associated protein 43
G-CSF	Granulocyte colony-stimulating factor
GFAP	Glial fibrillary acidic protein
GM-CSF	Granulocyte–macrophage colony-stimulating factor
GPx	Glutathione peroxidase
GSH	Glutathione
GST	Glutathione S-transferase
Gy	Gray
HDIR	High-dose ionising radiation
HDMEC	Human dermal microvascular endothelial cells
HNSCC	Head and Neck Squamous Cell Carcinoma
HO-1	Heme oxygenase-1
HSP27/HSP70	Heat shock protein 27/Heat shock protein 70
IL-1β, IL-6, IL-10, IL-2β	Interleukin-1 beta/6/10/2 beta
iNOS	Inducible nitric oxide synthase
JAK1	Janus kinase 1
JNK	c-Jun N-terminal kinases
Keap1	Kelch-like ECH-associated protein 1
LDIR	Low-dose ionising radiation
LET	Linear energy transfer
LRRK2	Leucine-rich repeat kinase 2
MAPK	Mitogen-activated protein kinase
MCF-7	Breast cancer side population cells
MCP-1	Monocyte chemoattractant protein-1 (also known as CCL2)
MDA	Malondialdehyde
MFN1	Mitofusin-1
MIP-1α	Macrophage inflammatory protein-1α
MMR	Mismatch repair
MnSOD	Manganese superoxide dismutase (also known as SOD2)
MPTP	1-methyl-4-phenyl-1,2,3,6-tetrahydropyridine
mROS	Mitochondrial reactive oxygen species
mtDNA	Mitochondrial DNA
NDDs	Neurodegenerative diseases
NF-κB	Nuclear factor kappa-light-chain-enhancer of activated B cells
NGF	Nerve growth factor
NHF	Normal human fibroblasts
Notch1	Neurogenic locus notch homolog protein 1
NPY	Neuropeptide Y
NQO1—NAD(P)H	Quinone oxidoreductase 1—NAD(P)H
Nrf2	Nuclear erythroid 2-related factor 2
NSCLC	Non-small-cell lung cancer
p53	Tumor protein p53
PARK2	Parkin RBR E3 ubiquitin protein ligase (PRKN, PARKIN)
PARP1	Poly [ADP-ribose] polymerase 1
PCP4	Purkinje cell protein 4 (also known as PEP-19)
PD	Parkinson’s disease
PET	Positron emission tomography
PI3K	Phosphoinositide 3-kinase
PIAS	Protein inhibitor of activated STAT
Prdx2	Peroxiredoxin-2
pTDP43	Phosphorylated TAR DNA-binding protein 43
ROS	Reactive oxygen species
SOCS	Suppressor of cytokine signaling
SOD	Superoxide dismutase
SP cells	Side population cells
STAT1/STAT3	Signal transducer and activator of transcription 1/3
STING	Stimulator of interferon genes
STMN2	Stathmin-2 (also known as SCG10)
Sv	Sievert
TGF-β	Transforming growth factor-beta
TNF-α	Tumor necrosis factor-alpha
TREM2	Triggering receptor expressed on myeloid cells 2
VEGF	Vascular endothelial growth factor
